# Thin layer drying models and characteristics of scent leaves (*Ocimum gratissimum*) and lemon basil leaves (*Ocimum africanum*)

**DOI:** 10.1016/j.heliyon.2021.e05945

**Published:** 2021-01-13

**Authors:** N.N. Mbegbu, C.O. Nwajinka, D.O. Amaefule

**Affiliations:** Department of Agricultural and Bioresources Engineering, Nnamdi Azikiwe University, Awka, Nigeria

**Keywords:** Thin-layer drying, Drying characteristics, Drying models, Scent leaves, Lemon basil leaves

## Abstract

The effect of air temperature on the drying kinetics and characteristics of scent and lemon basil leaves were investigated using a vacuum oven dryer at 30 °C, 40 °C, 50 °C, 60 °C and 70 °C. Suitable drying models were obtained to describe the drying process. Falling rate drying phenomenon was observed for both leaves. Six thin layer drying models namely: Lewis model, Page model, Modified Page model, Logarithmic model, Two term model and Midilli model were fitted to the moisture ratio data for both scent and lemon basil leaves. Highest coefficient of determination (R^2^), and least sum of square error (SSE) and root mean square error (RMSE) values were determined for the Logarithmic model for scent leaf as 0.9998, 0.0002, 0.0081, and 0.9961, 0.0034 and 0.0222 for lemon basil leaf. The Two term model showed the same values for scent leaf as the Logarithmic model and 0.9967, 0.0024 and 0.0185 for lemon basil leaf. They were the best fit models for all the drying temperatures. The results showed that scent and lemon basil leaves can best be dried at 70 °C and 60 °C, respectively. The specific energy consumption and the effective moisture diffusivities $\left(D_{eff}\right)$ of scent and lemon basil leaves were determined at different drying air temperatures. $D_{eff}$ranged from $4.76\;\times \;10^{-13}$to $1.47\;\times \;10^{-12}$m^2^/s and $4.80\;\times \;10^{-13}$ to $2.06\;\times \;10^{-12}$ m^2^/s for scent and lemon basil leaves respectively, as temperature increased. Using the Arrhenius equation, the activation energy $\left(E_{a}\right)$ and pre-exponential factor $\left(D_{o}\right)$ were determined as 25.01 kJ/mol and $8.19\;\times \;10^{-9}$ m^2^/s for scent leaf and 32.35 kJ/mol and $1.49\;\times \;10^{-7}$ for lemon basil leaves. Therefore, the Logarithmic and Two term models are recommended as the best models for the drying kinetics of scent and lemon basil leaves from the experiment.

## 1 Introduction

Vegetables provide bulk fibre, which aids digestion and are good sources of vitamins and minerals (Mohammadu et al., 2014), like ascorbic acid, beta carotene, riboflavin, Calcium, Magnesium, Iron, Phosphorus, etc (Lawal et al., 2015). They have been used from time as spices in food, condiments and for medicinal purposes like other plant materials (Nweze and Eze, 2009).

*Ocimum gratissimum*, popularly known as scent leaf is a fully developed flowering plant that is propagated by seed or cuttings. It grows throughout the tropics and subtropics but mostly in tropical Africa and India with great variability (Rhoda and Negimote, 2015). It contains alkaloids, saponins, terpenoids, flavonoids, tannins, phlobatannins, anthraquinones, steroids, and cardiac glycosides (Ijeh et al., 2004) and minerals like Calcium, Chloride, Manganese, Magnesium, Zinc and Potassium. The flowers and the leaves are rich in essential oil, and are used in the preparation of teas and infusion. *Ocimum gratissimum* is used in many countries for the traditional treatment of diarrhea, headache, fever, ophthalmic disorders, skin disease and pneumonia. The root is used as a pediatric sedative in the Brazilian forest areas (Njoku et al., 2011a, Njoku et al., 2011b). The plant is used in the treatment of epilepsy, high fever and diarrhea in the coastal areas of Nigeria and mental illness in the Savannah areas (Rhoda and Negimote, 2015). It is also used in the treatment of fungal infections, cold and catarrh, while the Igbos of South Eastern Nigeria employ it in the management of the neonatal umblicus and to keep wound surfaces sterile (Ijeh et al., 2004).

*Ocimum africanum*, known as lemon basil leaf belongs to the *Rutaceae* family, and is popularly called curry leaf in Nigeria. It is grown in tropical and subtropical regions, but is native to India. It can grow up to 20–40 cm (8–20 in) and bears white flowers in March to early April. Lemon basil leaf has been incorporated into alcoholic beverages (Hiltunen and Holm, 1999) and in many culinary preparations for ages; dishes, sauces, condiments, soups, stews, stuffing, teas, oils and cheeses. It is easily blended with other herbs, including, garlic, oregano, mustard, parsley, pepper, rosemary and thyme.

Green leafy vegetables are cultivated year-round in Nigeria but have peak period of considerable abundance. They are highly perishable and therefore require careful processing in order to preserve the nutrients, especially the water soluble vitamins. Drying minimizes handling and packaging requirements, and is a commonly used food preservation method (Oladele and Jimoh, 2017), but the products’ qualities are strongly dependent on the technique and the process variables (Oladosu-Ajayi et al., 2017) and the nature of the food material*.* Moisture content of food is reduced with an increased shelf life through convective and conductive drying mechanisms. The quality and quantity of an effectively dried food material determine its storability over a specific period of time depending on the nature of the food material. Traditionally, developing countries mostly practice open sun drying for food preservation, but vulnerability to weather, contamination with foreign matter, long process time, inconsistent product quality and low output are some of the associated problems (Rhoda and Negimote, 2015: Oladele and Jimoh, 2017).

Researchers have adopted other drying techniques as alternative to overcome these problems. Vacuum drying is exceptionally the ideal method for drying thermal and oxygen sensitive materials like vegetables due to the advantage of removing moisture at low temperature while minimizing reactions. In addition, effective hydraulic conductivity of material increases under vacuum, so the resistance to mass transfer at the product surface reduces.

Mathematical modeling of the drying process allows engineers to determine the most suitable operating conditions and to redesign drying equipment and chamber appropriately. Falling rate period is the period subsequent to the critical point and is achieved when the droplet surface is not saturated anymore, and drying is controlled by internal mass transfer of the volatile solvent to the surface. Beyond the falling period, the surface temperature rises, and the drying rate falls off rapidly. There are many models for simulation of thin layer drying processes namely: Lewis model, Page model, Modified Page model, Logarithmic model, Midilli model, Two-term model, etc.

Effective moisture diffusivity $\;\left(D_{eff}\right)$ varies with internal conditions such as the moisture content, temperature and the product's structure (Fernandez et al., 2018). Diffusivity kinetic models describes the drying phenomenon, optimizes the process and validates the model hypothesis based on the boundary and thermal conditions, physical variables or constants and product geometry. The interaction of moisture diffusivity, temperature and mass transfer during drying has been studied using Fick's diffusion model and Arrhenius equation by many researchers. Rhoda and Negimote (2015) studied the drying characteristics and kinetics of bitter leaf (*Vernonia amygdalina*) and scent leaf using a moisture content analyzer and reported their effective moisture diffusivity range as $8.437\;\times \;10^{-15}$ to $2.96\;\times \;10^{-12}$ m^2^/s and $8.67\;\times \;10^{-14}$to $4.51\;\times \;10^{-13}$ m^2^/s respectively. Esmaeel (2015) obtained a moisture diffusivity range of 1.624 × 10^−10^ to 7.652 × 10^−10^ m^2^/s for basil leaf drying with microwave dryer. Doymaz (2011) studied the drying of green bean and okra under solar energy and reported $D_{eff}$ of $1.12\;\times \;10^{-10}$m^2^/s and $1.52\times \;10^{-11}$ m^2^/s for green bean and okra, respectively. Other researches on fruits and vegetables drying include those on thyme leaves (Turan and Firatligil, 2019), apricots (Mirzaee et al., 2009), cocoa (Hii et al., 2009) and elephant apple (Nag and Dash, 2016). Effective diffusivity - absolute temperature relationship follows the first order rate process described by Arrhenius equation and can be characterized by the activation energy. Thus, the knowledge of activation energy and effective moisture diffusivity are necessary for designing and modeling the mass transfer processes (Fernandez et al., 2018).

A number of studies on drying of fruits and vegetables have been reported by various researchers from different regions of the world (Chinweuba et al., 2016: Oladele and Jimoh, 2017: Mirzaee et al., 2009: Doymaz, 2011: Esmaeel, 2015). To the best of the author's knowledge, study on the thin layer kinetics and characteristics of lemon basil leaf and scent leaf using vacuum oven dryer has not been reported yet. Therefore, the present research work ascertains the drying characteristics and kinetics of scent and lemon basil leaves using vacuum oven dryer, develops thin layer models for their drying processes and determines the moisture diffusivity, activation energy and pre-exponential factors needed for their drying equipment designs.

## 2 Materials and method

**Figure 1:**
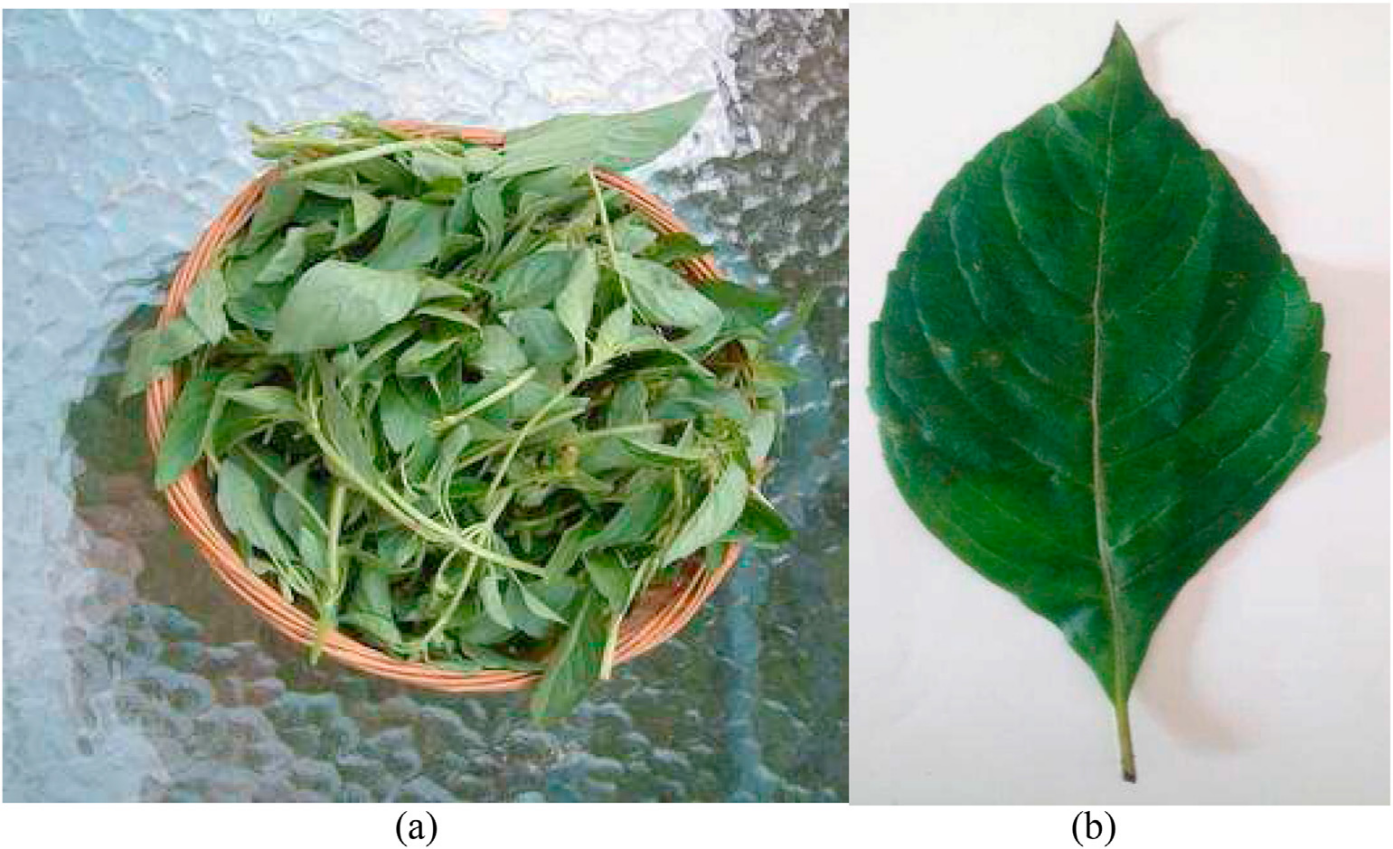
(a) Fresh lemon basil leaves (b) Fresh scent leaf.

Fresh samples of scent leaves and lemon basil leaves of peak flavor were purchased from the local market at Nsukka, Enugu state of Nigeria, and taken immediately to the crop science laboratory, Faculty of Agriculture, University of Nigeria Nsukka, Enugu State for identification and analysis. The samples were detached from the stems and washed thoroughly. The initial moisture of the samples were determined by oven drying at 105 °C for 24 h; as described by the Association of Official Analytical Chemists (AOAC) moisture content determination method (AOAC, 1990). Four samples of 30g each of scent leaves and lemon basil leaves were dried in an oven at the temperature 105 °C for 24 h (see Figure 1).

### 2.1 Experimental procedure

A laboratory vacuum oven dryer (Faithful oven DZ-3BE, China) with a technical specification of 220V, 50Hz and 2000W, RT + 10–250 °C and a vacuum degree of <133Pa, was used for the drying experiments at temperature levels of 30 °C, 40 °C, 50 °C, 60 °C and 70 °C. Fresh samples of scent leaves and lemon basil leaves were uniformly distributed in a thin layer on the oven tray. The moisture losses in the samples were measured by weight loss periodically at interval of 10 min for the 60 °C and 70 °C drying temperature and 30 min interval for the 30 °C, 40 °C and 50 °C temperature levels during the drying process to determine the drying rate. The experiments were conducted in three replicates of each treatment according to the respective temperatures. The drying process was continued until consecutive constant mass of the samples were observed. The moisture contents of the samples at any given time during the drying process were calculated using Eq. (1).

(1) $$M_{t}=\frac{m_{w}-m_{d}}{m_{d}}$$

where, $M_{t}$ is the moisture content (kg water/kg dry matter), $m_{w}$ is the wet mass of sample (kg) at time, t, (seconds) and $m_{d}$ is the dry mass of the sample (kg).

### 2.2 Drying curves

The moisture content decreases via moisture diffusion mechanism; it was described by Fick's second law of diffusion as a function of moisture gradient. The moisture content was converted to dimensionless moisture ratio (MR) as presented in Eq. (2).

(2) $$MR=\frac{M_{t}-M_{e}}{M_{o}-M_{e}}$$

where, $M_{t}$, $M_{e}$ and $M_{o}$ are moisture content at time t, equilibrium moisture content and initial moisture content respectively (kg water/kg dry mater).

For drying model selection, experimental data were fitted to six commonly known thin layer drying models namely: Lewis model, Page model, Modified Page model, Logarithmic model, Two term model and Midilli model, which are generally applicable for drying of fruits and vegetables. The regression analyses were carried out to determine these parameters: coefficient of determination (R^2^), sum of square error (SSE) and root mean square error (RMSE). The highest R^2^ values and least SSE and RMSE values indicate the best fit model (Turan and Firatligil, 2019; Nag and Dash, 2016). These parameters were evaluated with Equations (3), (4), (5) .

(3) $$R^{2}=\;\frac{{\left(\sum MR_{exp}\;\times \;MR_{pre}\right)}^{2}}{\sum MR_{exp}^{2}\;\times \;\sum MR_{pre}^{2}}$$

(4) $$RMSE={\left[\frac{1}{N}\overset{N}{\underset{i=1}{\sum }}{\left(MR_{exp,i}-MR_{pre,i}\right)}^{2}\right]}^{2}$$

(5) $$SSE=\;\overset{N}{\underset{i=1}{\sum }}{\left(MR_{exp,i}-MR_{pre,i}\right)}^{2}$$

where, N is number of observation, nis number of constants in the model, $MR_{exp,i}$ is experimental moisture ratio for the $i^{th}$observation, $MR_{pre,i}$ is predicted moisture ratio at the $i^{th}$observation.

### 2.3 Calculation of effective moisture diffusivity and activation energy

Diffusion of moisture in solids during drying is a complex process made up of surface diffusion, capillary flow and molecular diffusion which generally takes place in the falling rate period. These phenomena are combined into one term and represented with a lumped parameter called effective moisture diffusivity$\;\left(D_{eff}\right)$. In practice, an oven dryer for food materials is considerably more complex than a device that merely removes water, therefore, appropriate models are necessary for process design, energy integration, optimization and control. The first term of the Fick's diffusion equation is used to calculate the effective moisture diffusivity for a slab as illustrated in Eq. (6).

(6) $$MR=\frac{8}{\pi^{2}}\exp\left(-\;\frac{\pi^{2}D_{eff}}{4{\left(h^{*}\right)}^{2}}\right)\times \;t$$

where, $MR,\;D_{eff},\;and\;t$ were as previously defined and $h^{*}$ is the half thickness of slab (m) respectively. The leaves were assumed to be slab for the Fick's diffusion equation solution.

In order to obtain ($D_{eff})$, Eq. (6) was re-written in the straight-line form by taking logarithm on both sides yielding Eq. (7). The effective diffusivity was determined using the method of slopes of Eq. (7). By plotting the Ln(MR) against *t* from drying experiment data as shown in Figures 7 and 8 for scent and lemon basil leaves respectively, the slope which equals $-\frac{\pi^{2}D_{eff}}{4{\left(h^{*}\right)}^{2}}$ is evaluated for each of the drying temperatures.

(7) $$Ln\left(MR\right)=Ln\left(\frac{8}{\pi^{2}}\right)-\;\frac{\pi^{2}D_{eff}}{4{\left(h^{*}\right)}^{2}}\times \;t$$

Temperature has a significant effect on the effective diffusivity which can be described by the Arrhenius equation:

(8) $$D_{eff}=\;D_{0}\;\exp\left(-10^{3}\;\frac{E_{a}}{R\left(T+273.15\right)}\right)$$

where, $D_{0}$is Arrhenius factor (m^2^/s), $E_{a}$ is activation energy for diffusion (kJ/mol), R is universal gas constant = 8.314 (kJ/mol.K). T is temperature (K).

The activation energy was calculated from the slope of $Ln\left(D_{eff}\right)$ versus $\frac{1}{\left(T+273.15\right)}$ Eq. (9), which was plotted and presented on Figures 9 and 10 for scent and lemon basil leaves respectively. Eq. (9) was deduced from Eq. (8) above.

(9) $$Ln\left(D_{eff}\right)=\;Ln\left(D_{0}\right)-10^{3}\;\frac{E_{a}}{R\left(T+273.15\right)}$$

The slope of the plot of the Arrhenius equation is$\left(-10^{3}\;\frac{E_{a}}{R}\right)$, while the intercept is$\;Ln\left(D_{0}\right)$.

The drying process was terminated once no further reduction in weights of the samples was observed. Moisture content values were converted to moisture ratio and then fitted to the six thin layer drying models outlined in Table 1.

**Table 1:** Thin layer drying models applied to the drying of scent and lemon basil leaves.

Model name	Equation	References
Lewis	MR=exp(−kt)	Roberts et al. (2008)
Page	$MR=\text{exp}\left(-kt^{n}\right)$	Mundada et al. (2010)
Modified Page	$MR=\text{exp}{\left(-kt\right)}^{n}$	Yaldiz et al. (2001)
Logarithmic	MR=aexp(−kt)+c	Ertekin and Yaldiz (2004)
Two term	$MR=aexp\left(-k_{0}t\right)+bexp\left(-k_{1}t\right)$	Lee and Kim (2009)
Midilli	MR=exp(−kt)+bt	Midilli et al. (2002)

## 3 Results and discussions

**Figure 2:**
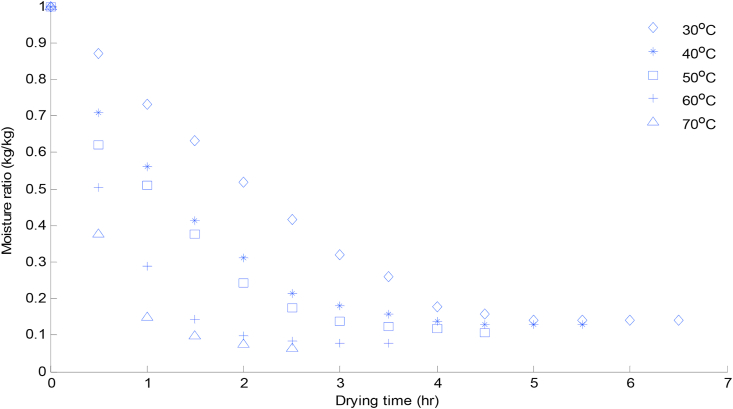
Moisture ratio change with time at different drying temperatures for scent leaves.

**Figure 3:**
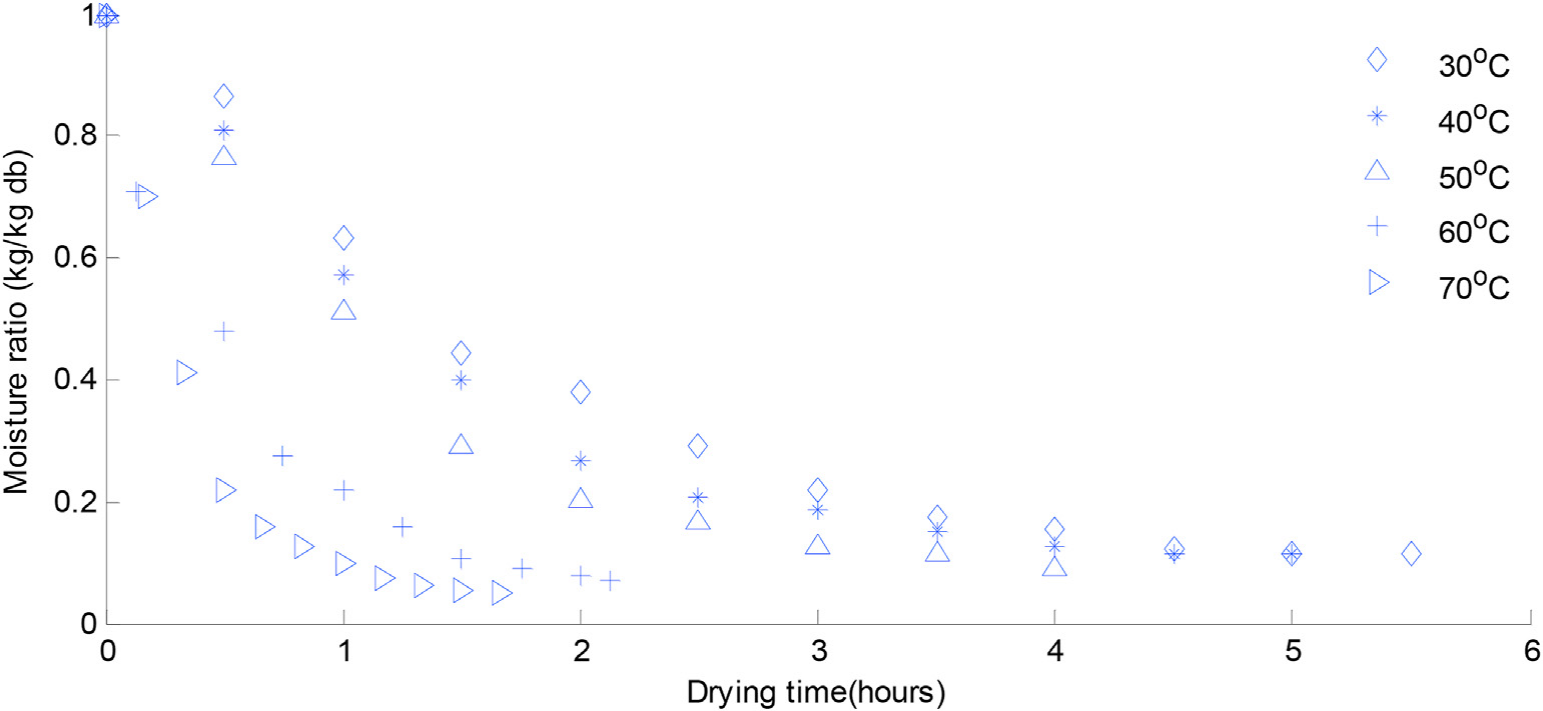
Moisture ratio change with time at different drying temperatures for lemon basil leaves.

The relationship between moisture ratio and drying time was shown in Figures 2 and 3 for scent leaf and lemon basil leaf respectively. Convective drying was not observed during the constant rate period. The moisture ratio decreased with increasing drying time during the falling rate period for the five different temperature levels in both leaves. This indicated that diffusion is the dominant physical mechanism for the moisture movement in the drying process (Onwude et al., 2016). The time taken to reduce the moisture content from the initial value of 79.80% (w.b) and 83.65% (w.b), to a final value of 0.2% and 0.1% at 30 °C drying temperature was; 6.5 h and 5.5 h for scent and lemon basil leaves respectively.

For both samples, the duration required to bring the samples to a constant moisture ratio was dependent on drying temperature; with highest duration recorded at 30 °C (6.5 h) scent leaf, (5.5 h) lemon basil and lowest at 70 °C (2.5 h) scent leaf and (99 min) for lemon basil leaf. These results were in agreement with the results of other researchers for various food materials such as scent leaf (Oladele and Jimoh, 2017), basil (Esmaeel, 2015), green bean and okra (Doymaz, 2011) thyme leaves (Turan and Firatligil, 2019) bitter leaf and scent leaf (Rhoda and Negimote, 2015) apricot (Mirzaee et al., 2010) and cocoa (Hii et al., 2009).

### 3.1 Mathematical modeling

The statistical analysis values obtained from fitting the experimental data to the widely used semi-theoretical thin layer models (mentioned in Table 1) are presented in Table 2. The R^2^, SSE and RMSE values ranged from 0.9573-0.9998, 0.0002–0.0389 and 0.0081–0.0623 for scent leaves and 0.9766–0.9967, 0.0024–0.0220 and 0.0185–0.0525 for lemon basil leaves, respectively. The values of R^2^, SSE and RMSE obtained with the Lewis's model for scent and lemon basil leaves were the same with that of Modified Page model. The lowest R^2^ values (0.9573) for scent and (0.9766) lemon basil leaves were observed at 40 °C with the Midilli model, while the least SSE values (0.0002) was observed at 70 °C with both Logarithmic and Two-term model for scent leaves and (0.0024) at 60 °C was observed with the Two-term model for lemon basil leaves. All the drying models indicated lower SSE and RMSE values at high temperature (60^o^C–70 °C).

**Table 2:** Statistical parameters for moisture ratio change models fit for both leaves samples drying.

Thin layer model	Sample	Temperature (^o^C)	R^2^	SSE	RMSE
Lewis model	Scent leaf	30	0.9870	0.0151	0.0341
		40	0.9686	0.0286	0.0488
		50	0.9738	0.0215	0.0463
		60	0.9862	0.0102	0.0382
		70	0.9890	0.0072	0.0382
	Lemon basil leaf	30	0.9887	0.0115	0.0323
		40	0.9856	0.0135	0.0367
		50	0.9872	0.0108	0.0368
		60	0.9938	0.0055	0.0249
		70	0.9890	0.0107	0.0328
Page model	Scent leaf	30	0.9904	0.0109	0.0302
		40	0.9863	0.0125	0.0337
		50	0.9901	0.0077	0.0294
		60	0.9918	0.0045	0.0275
		70	0.9933	0.0024	0.0247
	Lemon basil leaf	30	0.9894	0.0112	0.0336
		40	0.9857	0.0135	0.0387
		50	0.9881	0.0101	0.0379
		60	0.9939	0.0055	0.0262
		70	0.9890	0.0108	0.0348
Modified page model	Scent leaf	30	0.9870	0.0151	0.0355
		40	0.9686	0.0286	0.0510
		50	0.9738	0.0215	0.0489
		60	0.9862	0.0102	0.0412
		70	0.9890	0.0072	0.0427
	Lemon basil leaf	30	0.9887	0.0115	0.0339
		40	0.9856	0.0135	0.0388
		50	0.9874	0.0109	0.0394
		60	0.9938	0.0056	0.0264
		70	0.9890	0.0108	0.0346
Logarithmic model	Scent leaf	30	0.9889	0.0129	0.0343
		40	0.9965	0.0032	0.0178
		50	0.9948	0.0067	0.0290
		60	0.9989	0.0008	0.0129
		70	0.9998	0.0002	0.0081
	Lemon basil leaf	30	0.9916	0.0086	0.0309
		40	0.9910	0.0085	0.0325
		50	0.9891	0.0092	0.0392
		60	0.9961	0.0034	0.0222
		70	0.9943	0.0056	0.0265
Two term model	Scent leaf	30	0.9917	0.0097	0.0311
		40	0.9979	0.0019	0.0145
		50	0.9921	0.0065	0.0304
		60	0.9993	0.0005	0.0114
		70	0.9998	0.0002	0.0082
	Lemon basil leaf	30	0.9939	0.0057	0.0261
		40	0.9933	0.0060	0.0283
		50	0.9879	0.0113	0.0454
		60	0.9967	0.0024	0.0185
		70	0.9880	0.0107	0.0409
Midilli model	Scent leaf	30	0.9889	0.0129	0.0342
		40	0.9573	0.0389	0.0623
		50	0.9920	0.0065	0.0286
		60	0.9993	0.0005	0.0103
		70	0.9998	0.0002	0.0087
	Lemon basil leaf	30	0.9920	0.0082	0.0302
		40	0.9766	0.0220	0.0525
		50	0.9896	0.0088	0.0383
		60	0.9965	0.0032	0.0213
		70	0.9938	0.0061	0.0270

**Figure 4:**
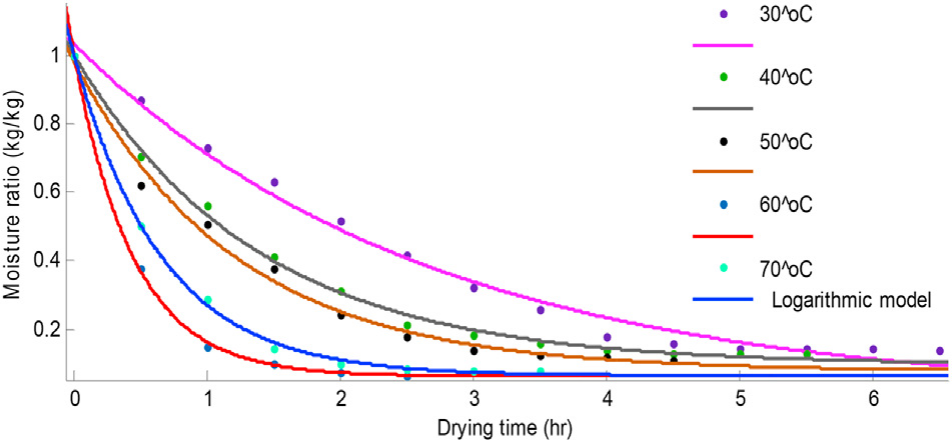
Comparison of experimental data and predicted moisture ratio using Logarithmic model for scent leaves.

**Figure 5:**
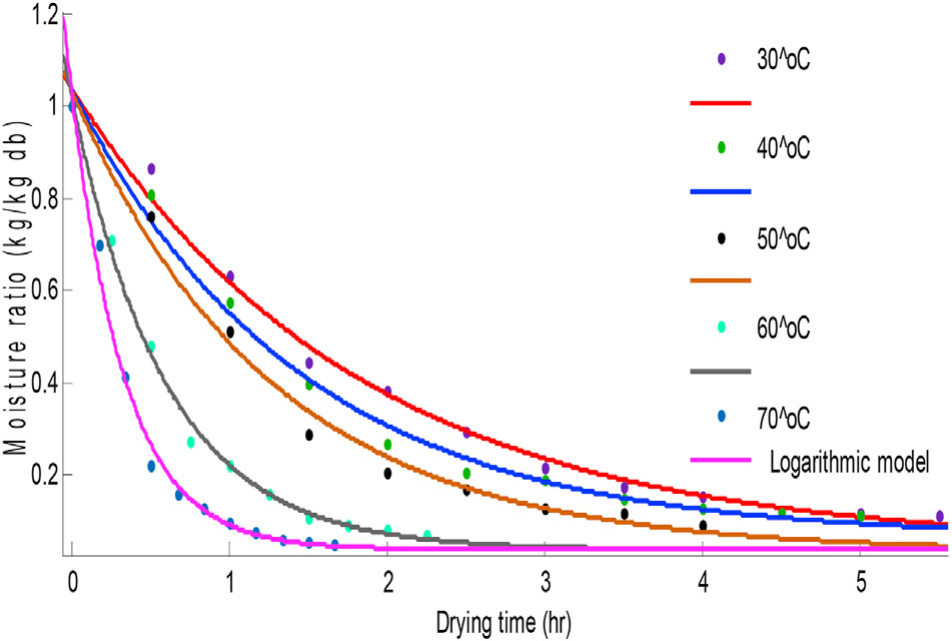
Comparison of experimental data and predicted moisture ratio using Logarithmic model for lemon basil leaves.

**Figure 6:**
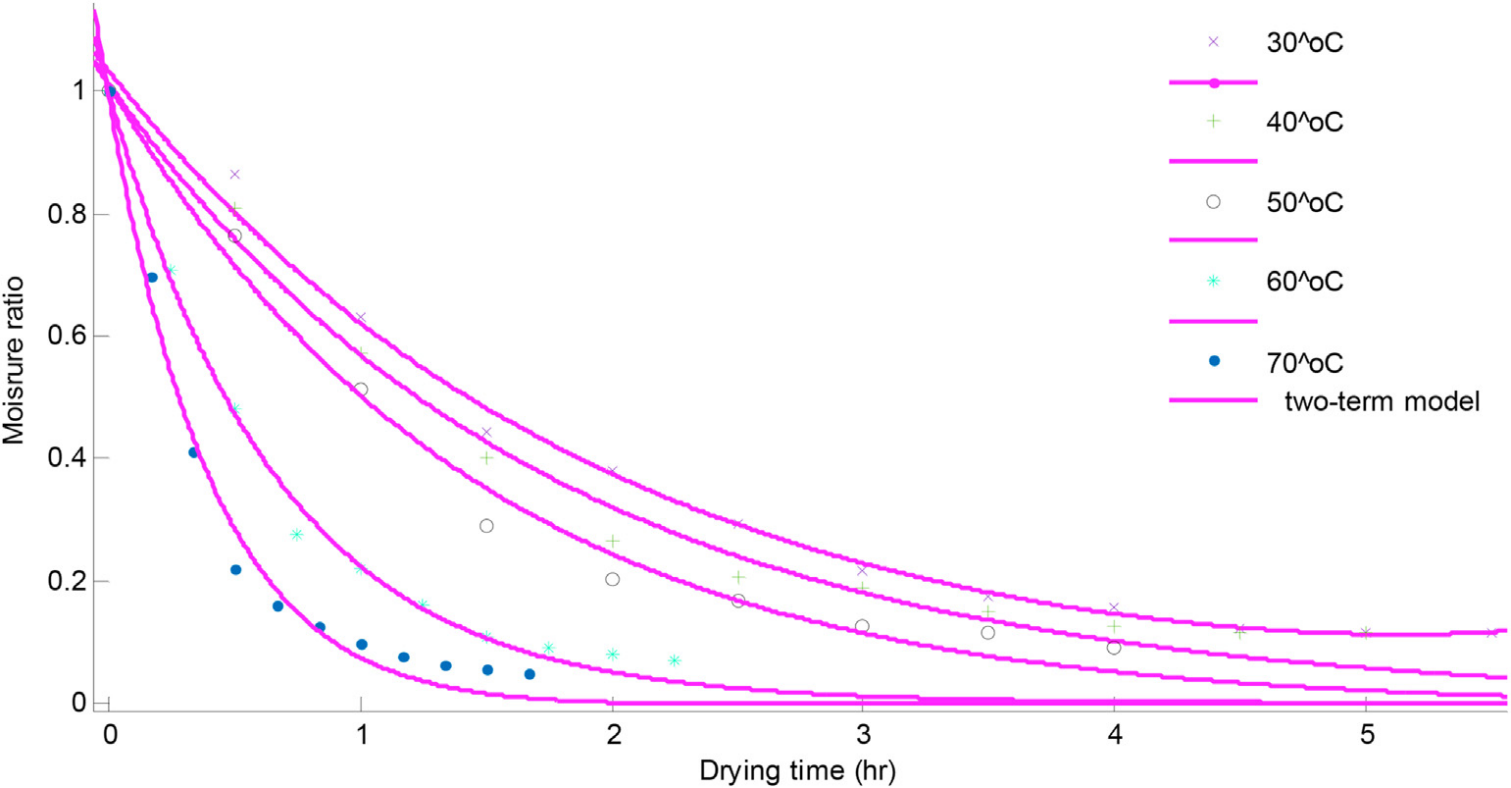
Comparison of experimental data and predicted moisture ratio using Two-term model for lemon basil leaves.

The values of R^2^ obtained from Logarithmic and Two-term models were slightly higher than the others as presented in Table 2. The R^2^ values were higher at higher temperatures (60^o^C–70 °C) for scent and lemon basil leaves. As earlier stated, the Logarithmic and Two-term models showed higher SSE values at lower temperatures (30^o^C–50 °C) for both scent and lemon basil leaves. The both models showed highest R^2^ values (0.9998) at 70 °C for scent leaves and (0.9967) at 60 °C for lemon basil leaves. Hence, scent and lemon basil leaves can best be dried at 70 °C and 60 °C respectively. Comparisons of the predicted values of the Logarithmic model with the experimental data are shown on Figures 4 and 5 for scent and lemon basil leaves respectively. Also, the graph of predicted values of moisture ratios obtained from the Two-term model for lemon basil leaves is shown in Figure 6. It can be seen from Figures 4, 5, and 6 that both Logarithmic and Two-term models show good fits to the experimental values. The Two-term model shows better fit at lower temperatures while the Logarithmic model shows better fit at higher temperatures for lemon basil leaves.

Report by Lee and Kim (2009) showed that the Logarithmic model showed a better fit when compared to other models used in the drying kinetics of Asian white radish slices. Turan and Firatligil (2019) reported that the Logarithmic model showed good fit at lower temperatures (50 °C–60 °C) for thyme (*Thymus vulgaris*) leaves drying using conventional oven. Esmaeel (2015) reported that the Logarithmic model showed good fit for modeling basil leaves drying with microwave at different microwave powers. Both the Logarithmic and the Midilli models were found to be suitable for the drying of apricot at air velocity of 1 m/s and 2 m/s, respectively (Mirzaee et al., 2010).

### 3.2 Effective moisture diffusivity and activation energy

**Figure 7:**
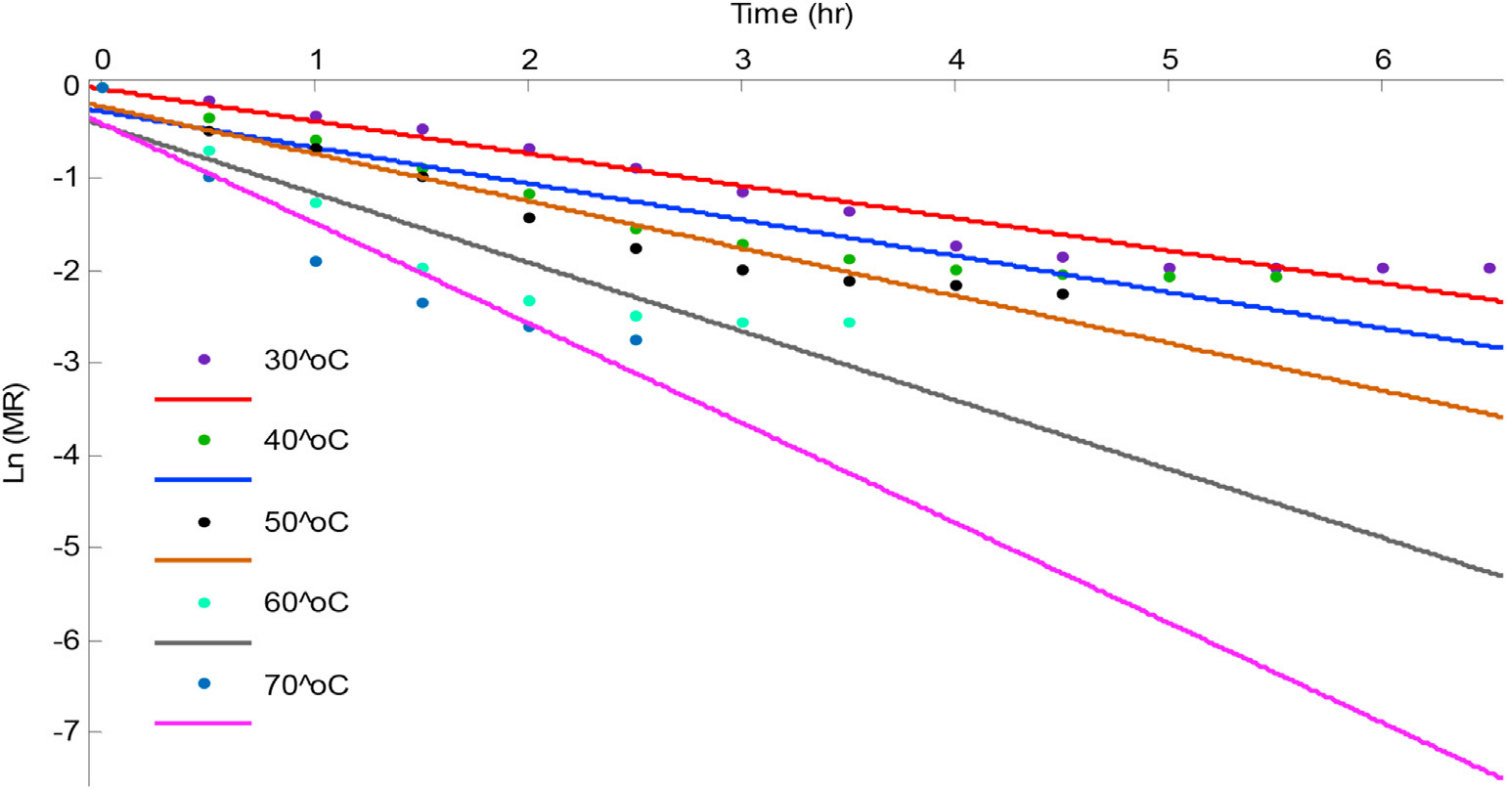
A plot of Logarithmic moisture ratio versus drying time for scent leaves.

**Figure 8:**
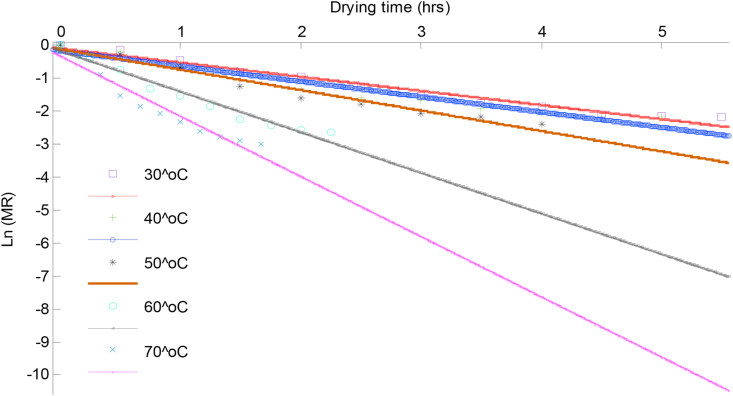
A plot of Logarithmic moisture ratio versus drying time for lemon basil leaves.

The effective moisture diffusivity for scent and lemon basil leaves at different temperatures based on Fick's second law of diffusion (Equation 9) were derived from the plot of Ln(MR) versus time in Figures 7 and 8 and are presented in Table 3.

**Table 3:** Estimated effective moisture diffusivity of samples and their models predictive fit parameter.

Samples	Drying air Temperature (^o^C)	Slope	$D_{\mathrm{eff}}$ (m^2^/s)	R^2^
Scent leaves	30	-0.3501	4.76E-13	0.9504
	40	-0.3504	4.77E-13	0.8867
	50	-0.4598	6.26E-13	0.9203
	60	-0.7442	1.01E-12	0.8785
	70	-1.0800	1.47E-12	0.8992
Lemon basil leaves	30	-0.4922	4.83E-13	0.9726
	40	-0.4660	5.24E-13	0.9495
	50	-0.6217	7.00E-13	0.9637
	60	-1.2330	1.39E-12	0.9670
	70	-1.8270	2.06E-12	0.9431

The curves were fitted to straight lines with R^2^ values of 0.8785–0.9726 which showed that liquid diffusion is the driving force regulating the drying process. The values of effective moisture diffusivity were found to be in the range of 4.76 × 10^−13^ to 1.47 × 10^−12^ m^2^/s and 4.8 × 10^−13^ to 2.06 × 10^−12^ m^2^/s for scent and lemon basil leaves respectively with increasing temperature. The effective moisture diffusivity of lemon basil leaves were slightly higher than the values of scent leaves, this could be because of the slight difference in the thickness of the lemon basil (1.0 × 10^−4^ m) compared to scent leaves (1.1 × 10^−4^ m). Comparable values of $D_{eff}$ have been reported by other investigators; 8.6 × 10^−14^ to 4.5 × 10^−13^ m^2^/s for scent leaves at 50–80 °C (Rhoda and Negimote, 2015) and 2.59 × 10^−9^ to 1.28 × 10^−8^ m^2^/s for thyme leaves at 50–80 °C (Turan and Firatligil, 2019).

Doymaz (2011) reported $D_{eff}$ of 1.12 × 10^−10^ and 1.52 × 10^−11^ m^2^/s for green bean and okra respectively. The maximum R^2^ values obtained in the present study were 0.9504 and 0.9726 at drying temperature of 30 °C for scent and lemon basil leaves respectively, while the minimum value of 0.8785 at 60 °C for scent leaves and 0.9431 at 70 °C for lemon basil leaves.

**Figure 9:**
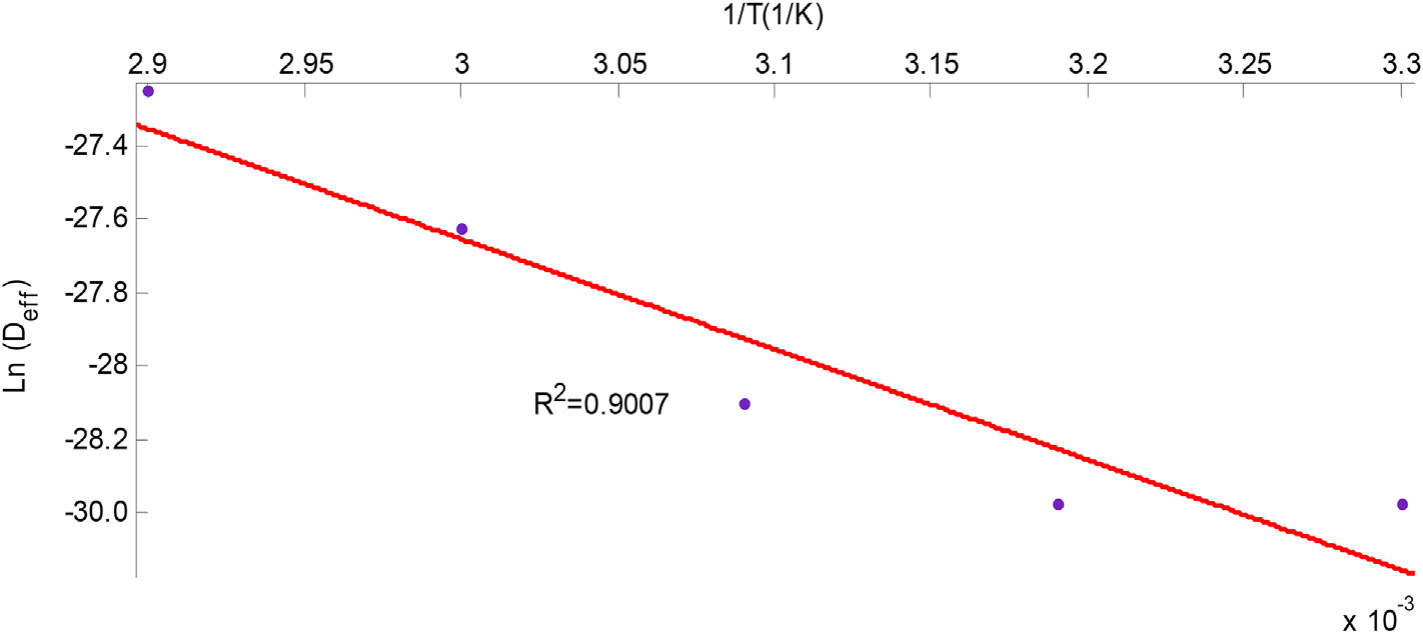
Influence of temperature on the effective diffusivity of scent leaves.

**Figure 10:**
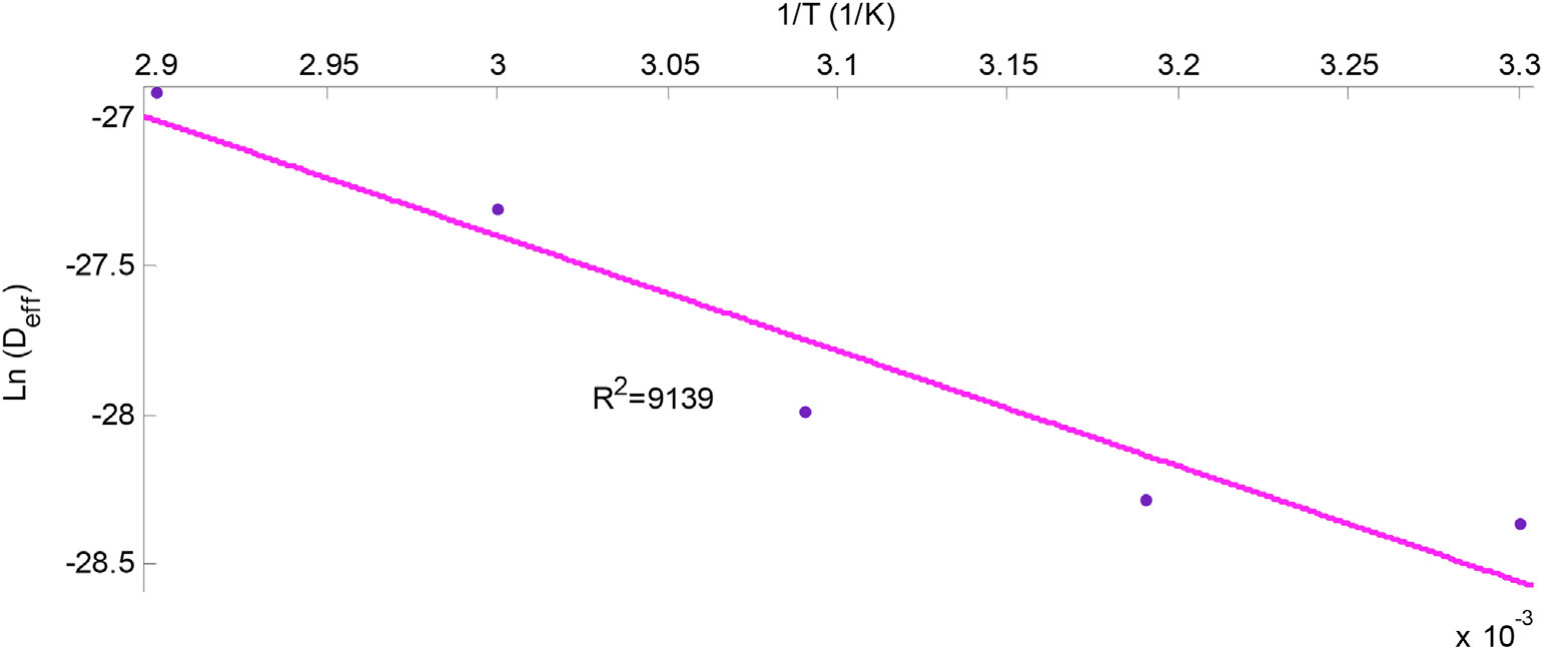
Influence of temperature on the effective diffusivity of lemon basil leaves.

The plot of $Ln\;\left(D_{eff}\right)$ against $\frac{1}{T}$ is shown in Figure 9 for the scent leaf and Figure 10 for the lemon basil leaf. The reliance of effective moisture diffusivity on temperature can be seen; the straight line graph indicates Arrhenius dependency (Equation 10). The activation energy $\left(E_{a}\right),$ was estimated from the graph to be 25.01 kJ/mol and 32.35 kJ/mol for scent and lemon basil leaves, while their Arrhenius constants$\;\left(D_{o}\right)$, were 8.19 × 10^−9^ and 1.49 × 10^−7^ m^2^/s respectively. Lower activation energy indicates lower sensitivity to air temperature. Values of activation energy for most agricultural food products lie within the range of 12.7–110 kJ/mol (Akpinar et al., 2003; Bablis and Belessiotis, 2004). The activation energies in comparison with other food materials reported in literature is presented in Table 4. The ${\text{E}}_{\text{a}}$ obtained from this work for scent leaf is found to be in close range with other food products reported such as bitter leaf (53.55 kJ/mol) (Rhoda and Negimote, 2015), scent leaves (31.801 kJ/mol) (Oladele and Jimoh, 2017) and cocoa (44.92 kJ/mol) (Hii et al., 2009).

**Table 4:** Activation energy of scent and lemon basil leaves and other products.

Material	Activation Energy Ea (kJ/mol)	References
Scent leaf	25.01	Present work
Lemon basil leaf	32.34	Present work
Bitter leaf	53.55	Rhoda and Negimote (2015)
Red chilli	41.95	Gupta et al. (2002)
Scent leaf	31.80	Oladele and Jimoh (2017)
Elephant apple	21.95	Nag and Dash (2016)
Mint	82.93	Park et al. (2002)
Basil	33.21	Kadam et al. (2011)
Thyme leaves	21.40	Turan and Firatligil (2019)

## 4 Conclusion

The drying characteristics of scent and lemon basil leaves were investigated at drying air temperatures of 30 °C, 40 °C, 50 °C, 60 °C and 70 °C using a vacuum oven dryer. The drying process occurred in falling rate period but was not observed in constant rate period. The experimental data were fitted into the six thin layer drying models and goodness of fit determined using R^2^, SSE and RMSE. According to the results, the Logarithmic and Two-term models could adequately describe the thin layer drying behaviour of scent and lemon basil leaves. The most appropriate temperature for drying of scent and lemon basil leaves was found to be 70 °C and 60 °C, respectively. The effective moisture diffusivity values were estimated from Fick's diffusion model and vary from 4.76 × 10^−13^ to 1.47 × 10^−12^ m^2^/s and 4.8 × 10^−13^ to 2.06 × 10^−12^ m^2^/s for scent and lemon basil leaves respectively. Increase in drying temperature led to a resultant increase in effective moisture diffusivity. The activation energy, $\left(E_{a}\right)$ values obtained using the Arrhenius equation for scent and lemon basil leaves were 25.01 kJ/mol and 32.35 kJ/mol while their corresponding Arrhenius constants $\left(D_{o}\right)$ were 8.19 × 10^−9^ m^2^/s and 1.49 × 10^−7^ m^2^/s respectively. The results of this study are useful to optimize drying process parameters for commercial scale production of dried scent and lemon basil leaves using vacuum oven dryer and to achieve superior quality of the dried products.

## Declarations

### Author contribution statement

Mbegbu, N. N.: Conceived and designed the experiments; Performed the experiments; Analyzed and interpreted the data; Contributed reagents, materials, analysis tools or data; Wrote the paper.

Nwajinka, C. O. Analyzed and interpreted the data; Contributed reagents, materials, analysis tools or data.

Amaefule, D.O.: Analyzed and interpreted the data.

### Funding statement

This research did not receive any specific grant from funding agencies in the public, commercial, or not-for-profit sectors.

### Data availability statement

Data will be made available on request.

### Declaration of interests statement

The authors declare no conflict of interest.

### Additional information

No additional information is available for this paper.
